# Variable Landscape of PD-L1 Expression in Breast Carcinoma as Detected by the DAKO 22C3 Immunohistochemistry Assay

**DOI:** 10.1093/oncolo/oyad025

**Published:** 2023-03-02

**Authors:** Natalie Danziger, Ethan S Sokol, Ryon P Graf, Matthew C Hiemenz, Jake Maule, Vamsi Parimi, Carlo Palmieri, Lajos Pusztai, Jeffrey S Ross, Richard S P Huang

**Affiliations:** Foundation Medicine, Inc., Cambridge, MA, USA; Foundation Medicine, Inc., Cambridge, MA, USA; Foundation Medicine, Inc., Cambridge, MA, USA; Foundation Medicine, Inc., Cambridge, MA, USA; Foundation Medicine, Inc., Cambridge, MA, USA; Foundation Medicine, Inc., Cambridge, MA, USA; Department of Molecular and Clinical Cancer Medicine, University of Liverpool, Liverpool, UK; The Clatterbridge Cancer Centre National Health Service (NHS) Foundation Trust, Liverpool, UK; Foundation Medicine, Inc., Cambridge, MA, USA; Departments of Pathology and Urology, State University of New York Upstate Medical University, Syracuse, NY, USA; Foundation Medicine, Inc., Cambridge, MA, USA

**Keywords:** breast cancer, immunotherapy, PD-L1, triple-negative breast cancer, biomarkers, comprehensive genomic profiling

## Abstract

**Background:**

In 2020, pembrolizumab was approved as a therapy for triple-negative breast cancer (TNBC) with the companion diagnostic DAKO 22C3 programmed death ligand-1 (PD-L1) immunohistochemistry assay. The study aimed to determine the landscape of PD-L1 expression as detected by the DAKO 22C3 PD-L1 assay in breast cancer subtypes and compare the clinicopathologic and genomic characteristics of PD-L1 positive and negative TNBC.

**Methods:**

PD-L1 expression using the DAKO 22C3 antibody was scored using a combined positive score (CPS) and positive status was defined as CPS ≥10. Comprehensive genomic profiling was performed using the FoundationOne CDx assay.

**Results:**

Of the 396 BC patients stained with DAKO 22C3, the majority were HR+/HER2− and TNBC (42% and 36%, respectively). Median PD-L1 expression and frequency of CPS ≥10 was highest in TNBC cases (median: 7.5, 50% CPS ≥10) and lowest in the HR+/HER2− group (median: 1.0, 15.5% CPS ≥10) (*P* < .0001). A comparison of PD-L1 positive and PD-L1 negative TNBC demonstrated no significant differences in clinicopathologic or genomic characteristics. TNBC tissue samples from the breast did have an observed enrichment for PD-L1 positivity compared to TNBC tissue samples from a metastatic site (57% vs. 44%), but this was not statistically significant (*P* = .1766). In the HR+/HER2− group, genomic alterations in *TP53*, *CREBBP*, and *CCNE1* were more prevalent and genomic loss of heterozygosity was higher in the PD-L1(+) group compared to the PD-L1(−) group.

**Conclusions:**

The subtypes of breast cancer have distinct patterns of PD-L1 expression, supporting that further research of immunotherapies may include specific evaluation of optimum cutoffs for non-TNBC patients. In TNBC, PD-L1 positivity is not associated with other clinicopathologic or genomic features and should be integrated into future studies of immunotherapy efficacy.

## Teaser

The objective of this study was to determine the landscape of PD-L1 protein expression in breast cancer using the DAKO 22C3 assay and to compare PD-L1–positive and PD-L1–negative cancers by comprehensive genomic profiling to determine if these populations differ in clinical or genomic characteristics.

**Implications for Practice**

This article presents a retrospective analysis of the patterns of PD-L1 expression using the DAKO 22C3 assay across all subtypes of breast carcinoma and describes the correlation of PD-L1 with other genomic features. The prevalence of PD-L1 expression varied by subtype. Within TNBC, PD-L1 positivity did not correlate with other clinicopathologic or genomic characteristics suggesting the independent nature of PD-L1 as a biomarker in TNBC. These conclusions support that further research on immunotherapies in non-TNBC patients may include specific evaluation of optimum PD-L1 scoring cutoffs.

## Introduction

Despite advances in early detection and treatment, breast cancer remains the second leading cause of cancer-related death for females in the USA.^1,2^ In routine clinical practice, breast cancers are classified based on the expression of estrogen receptor (ER), progesterone receptor (PR), and human epidermal growth factor receptor (HER2). In 15% of breast cancers, there is an absence of expression of these 3 receptors, so-called triple-negative breast cancer (TNBC).^3,4^ TNBC is characterized by aggressive pathologic features and high rates of distant recurrence. TNBC have the lowest 5-year survival rate among the receptor expression-defined subtypes.^1,3,5^ In addition, TNBC is more prevalent in African American women and contributes to the disparate outcomes for this population.^6,7^

Chemotherapy remains the standard of care first-line treatment for metastatic TNBC. Various anthracyclines, taxanes, and alkylating agents are commonly used in this setting.^1^ One of the challenges of treating *BRCA* wild-type TNBC is that it lacks targeted therapy options, so there is a strong dependence on cytotoxic agents. A new therapeutic option became available for patients in 2019 with the approval of the immune checkpoint inhibitor (ICPI) atezolizumab in combination with *nab*-paclitaxel for locally advanced or metastatic TNBC that is PD-L1 positive based on improved progression-free survival (PFS) observed in the IMpassion130 trial using the VENTANA SP142 PD-L1 assay.^8-10^ However, the indication was subsequently withdrawn in the USA in 2021 after additional studies did not show improvement in survival.^11,12^ During this time, another immunotherapy option became available for PD-L1 positive TNBC patients; pembrolizumab in combination with chemotherapy was approved in November, 2020, which was supported by the results of the KEYNOTE-355 study.^13^ Pembrolizumab and atezolizumab are ICPI targeting the programmed death receptor-1 (PD-1) and programmed death ligand-1 (PD-L1), respectively. PD-L1 is expressed by tumor-infiltrating leukocytes (TILs) in the tumor microenvironment and by tumor cells themselves as an adaptation to evade anti-tumor immune responses.^14,15^ Multiple studies showed that approximately half of TNBC express PD-L1, predominantly in TILs rather than tumor cells.^1,15-18^

In the KEYNOTE-355 trial (NCT02819518), 847 patients with untreated locally recurrent, inoperable, or metastatic TNBC received either pembrolizumab plus chemotherapy, or placebo plus chemotherapy with the physician’s choice of *nab*-paclitaxel, paclitaxel, or gemcitabine plus carboplatin for the chemotherapy regimen.^19^ Patients were assessed for PD-L1 expression by combined positive score (CPS) using the DAKO PD-L1 IHC 22C3 pharmDx assay.^20^ Among patients with a CPS of ≥10, median PFS was 9.7 months for patients receiving pembrolizumab versus 5.6 months for patients receiving placebo (hazard ratio [HR] 0.65, 95% CI, 0.49-0.86, *P* = .0012). In addition, PFS was evaluated at a CPS cutoff of ≥1 (7.6 vs. 5.6 months, HR 0.82, 95% CI, 0.74-0.90, *P* = .0014) and in the intent-to-treat population (7.5 vs. 5.6 months, HR 0.82, 95% CI, 0.69-0.97), but the threshold for significance based on the pre-specified statistical criteria was not met in either of these populations. These study results led to the FDA accelerated approval in November, 2020 of pembrolizumab plus chemotherapy for TNBC patients with a PD-L1 CPS ≥10. The benefit was confirmed in the survival analysis which reported overall survival (OS) of 23 months for pembrolizumab plus chemotherapy versus 16.3 months for placebo plus chemotherapy (HR 0.73, 95% CI, 0.55-0.95, *P* = .0093).^21^

Biomarkers predicting benefits from ICPI other than PD-L1 expression have also emerged in recent years. Both microsatellite instability-high (MSI-H) and tumor mutational burden ≥10 mutations per megabase (TMB-High) have also been approved as pan-solid tumor companion diagnostics for pembrolizumab.^22,23^ In addition, *CD274* gene (that encodes PD-L1), copy number alterations, mutations, and rearrangements are emerging as candidate biomarkers for benefit from ICPI.^24-27^ This study aimed to determine the landscape of PD-L1 protein expression in breast cancer using the DAKO 22C3 assay and to compare PD-L1 positive and PD-L1 negative cancers by comprehensive genomic profiling (CGP) to determine if these populations differ in clinical or genomic characteristics.

## Methods

### Patient Selection

All patients meeting the following criteria were included in this study: (1) CGP performed using the FoundationOne^®^CDx assay between November, 2018 and October, 2021; (2) a diagnosis of breast carcinoma as reported by the submitting physician and accompanying pathology report; and (3) PD-L1 IHC was evaluated by DAKO 22C3 assay and scored with CPS. Thus, this US national cohort consisted of patients from multiple institutions who submitted breast carcinoma specimens for CGP to Foundation Medicine during the specified time frame. ER, PR, and HER2 expression status were extracted from the documentation submitted as a part of the routine course of clinical testing. In cases where HER2 expression was not reported, detection of *ERBB2* amplification by CGP was used to classify the patient’s receptor status. Patients that were positive for ER and/or PR expression and negative for HER2 expression were classified as HR+/HER2−, patients that were positive for HER2 expression regardless of ER/PR status were classified as HER2+, and patients that were negative for all three biomarkers were classified as triple-negative breast carcinoma (TNBC).

### PD-L1 IHC

PD-L1 IHC was performed in a Clinical Laboratory Improvement Amendments (CLIA)-certified, College of American Pathologists (CAP)-accredited laboratory (Foundation Medicine, Morrisville, NC, USA). PD-L1 IHC was tested using the DAKO PD-L1 IHC 22C3 pharmDx assay which uses the mouse monoclonal 22C3 anti-PD-L1 clone. The assay was performed according to the package insert with appropriate controls.^20^ Scoring was performed by American Board of Pathology board-certified pathologists specifically trained in PD-L1 22C3 CDx scoring for TNBC. Borderline cases underwent review by a 2nd pathologist to come to a consensus for scoring. Scores are reported as a CPS which is the number of PD-L1 staining cells (including tumor cells, lymphocytes, and macrophages) divided by the total number of viable tumor cells and then multiplied by 100. A sample was considered PD-L1(+) when CPS was ≥10.

### Comprehensive Genomic Profiling

CGP was performed in a CLIA-certified, CAP-accredited laboratory (Foundation Medicine, Cambridge, MA, USA and Morrisville, NC, USA). Sequencing was performed using adaptor-ligation and hybrid capture from ≥50 ng DNA extracted from formalin-fixed paraffin-embedded samples as previously described.^28^ Exons from 324 genes and select introns from 36 genes were interrogated for all classes of genomic alterations (GA) (short variants, copy number changes, and rearrangements) by the assay.^29^*CD274* amplification was defined as a CN of ploidy +4 for the purposes of this study. Tumor mutational burden was assessed on 0.80 megabases (Mb) of sequenced DNA and calculated based on the number of somatic base substitution or insertion/deletion alterations per Mb after excluding known somatic and deleterious mutations as previously described.^30^ MSI was determined on 95 loci as previously described.^31^

As research uses only (RUO), genomic loss of heterozygosity (gLOH) and genetic ancestry were calculated. gLOH was calculated by quantifying the loss of heterozygosity at over 3 500 SNPs but excluding whole chromosome arm losses (>90% arm loss), a method that was described and validated in the ARIEL2 trial for ovarian cancer.^32,33^ Scores are reported as a percentage, and specimens were required to have ≥30% tumor nuclei to meet quality control for inclusion in the analysis. Predominant genetic ancestry was assessed using a SNP-based approach.^34^ Using the data from the 1000 Genomes set, a trained and validated classifier was developed based on over 40 000 germline SNPs that could be identified in both the 1000 Genomes data and the profiling assay used here. Individuals could be classified into one of five possible predominant ancestry groups: African (AFR), European (EUR), Central and South American (AMR), South Asian (SAS), and East Asian (EAS).

### Data Analysis

Statistics were evaluated using R version 3.6.1. Fisher’s exact test was used for categorical variables, and *P*-values were corrected using Benjamini–Hochberg corrections for multiple comparisons when appropriate. The Wilcox and the Kruskal–Wallis tests were used to assess continuous, non-parametric variables including TMB and age.

## Results

### Patient Characteristics

A total of 396 patients were identified for inclusion in this study. The HR+/HER2− subtype was the most prevalent (*n* = 168), followed by TNBC (*n* = 142), and HER2+ (*n* = 18). In addition, 68 cases had unknown receptor status. Nearly all patients were female (99.5%, 394/396), and they had a median age of 62 years (range 29-89) which was similar across all subtypes (Table 1). The majority of patients were of predominantly European ancestry (66.4%, 263/396), while the remainder were mostly of African and American ancestry (17.7% and 11.6%, respectively). Notably, there was a numerical enrichment of patients with African ancestry in the TNBC subtype compared to the HR+/HER2− subtype (23.2% vs. 14.3%, *P* = .055).

**Table 1.** Summary of patient characteristics molecular subtype using DAKO 22C3 PD-L1 IHC assay.

	HER2+ (*N* = 18)	HR+/HER2− (*N* = 168)	Triple-negative (*N* = 142)	Unknown (*N* = 68)	Total (*N* = 396)	*P*-value
Age, median [IQR] years	62.5 [55-68]	63 [52-69]	60 [51-69]	62.5 [52-74]	62	.588
Sex (% female)	17 (94.4%)	168 (100.0%)	142 (100.0%)	67 (98.5%)	394 (99.5%)	.024
Genetic ancestry						.027
AFR	2 (11.1%)	24 (14.3%)	33 (23.2%)	11 (16.2%)	70 (17.7%)	
AMR	1 (5.6%)	21 (12.5%)	15 (10.6%)	9 (13.2%)	46 (11.6%)	
EAS	5 (27.8%)	4 (2.4%)	3 (2.1%)	1 (1.5%)	13 (3.3%)	
EUR	10 (55.6%)	117 (69.6%)	89 (62.7%)	47 (69.1%)	263 (66.4%)	
SAS	0 (0.0%)	2 (1.2%)	2 (1.4%)	0 (0.0%)	4 (1.0%)	
MSI-H	0 (0%)	0 (0%)	0 (0%)	0 (0%)	0 (0%)	1
TMB, median [IQR] mut/MB	4.4 [2.5-6.3]	2.5 [1.3-5.0]	2.5 [1.3-5.0]	2.5 [1.3-6.3]	2.5	.064
PD-L1 CDx positive	8 (44.4%)	26 (15.5%)	71 (50.0%)	23 (33.8%)	128 (32.3%)	<.001
PD-L1 CPS, median [IQR]	5 [1-18]	1 [0-2]	7.5 [1-20]	1 [0-10]	1 [0-10]	<.001

Abbreviations: AFR, African; AMR, Central and South American; CPS, combined positive score; EUR, European; MSI-H, microsatellite instability-High; PD-L1, programmed death ligand-1; SAS, South Asian; TMB, tumor mutational burden.

### DAKO 22C3 PD-L1 Expression Patterns

**Figure 1.**
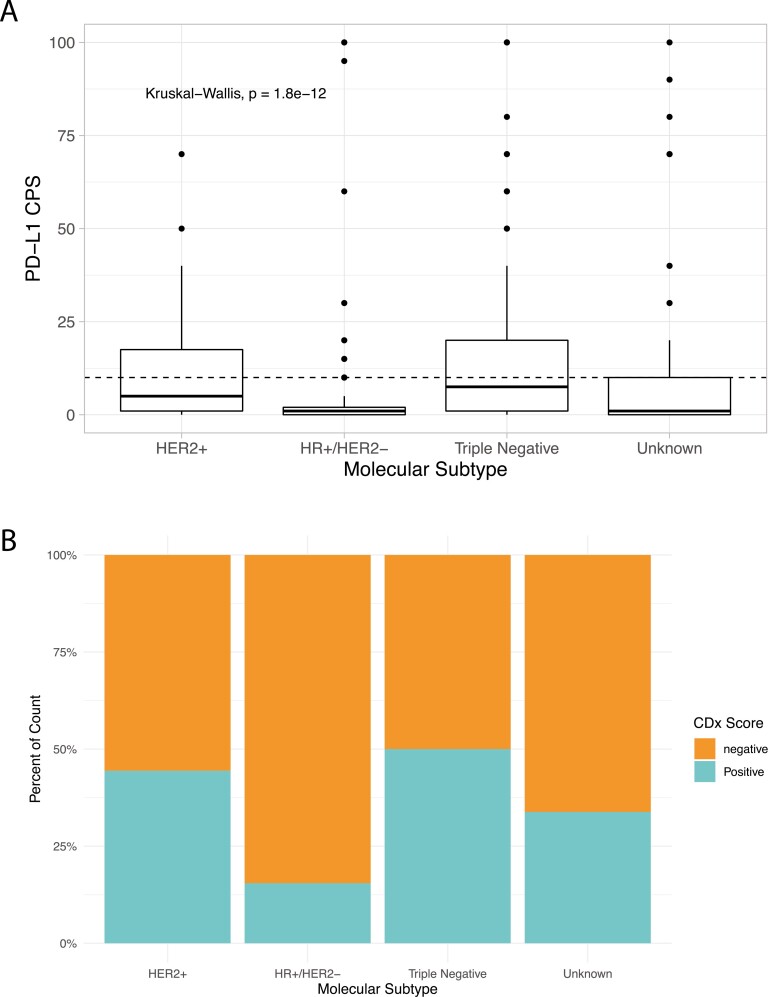
(**A**) PD-L1 immunohistochemistry (IHC) combined positive scores (CPS) using the Dako 22C3 assay for breast cancers by molecular subtype. (**B**) % of breast cancer cases with PD-L1 IHC CPS ≥10 by molecular subtype.

The overall PD-L1 positivity (CPS ≥10) rate was 32%. Median CPS for PD-L1 expression varied significantly by subtype (Fig. 1A). TNBC had the highest median CPS at 7.5 (IQR: 1.0-20) while HR+/HER2− had the lowest at 1.0 (IQR: 0-2.0) (*P* < .0001). TNBC also had greater median PD-L1 expression than the group of unknown subtype (CPS 1.0, IQR: 0-10.0) (*P* = .015), and the unknown subtype had greater median PD-L1 expression than the HR+/HER2− subtype (CPS 1.0 IQR: 0-2.0) (*P* = .0009). A similar trend was also observed when PD-L1 expression was classified as positive defined as a CPS ≥10 (Fig. 1B). TNBC had the highest rate of positivity at 50.0% (71/142) followed by the HER2+ group at 44.4%, (8/18) and the HR+/HER2− subset had the lowest rate of positivity at 15.5% (26/168).

### PD-L1 Positive versus PD-L1 Negative TNBC Cohort Based on DAKO 22C3 CDx Assay

We performed a comparison of PD-L1(+) (*n* = 71) and PD-L1(−) (*n* = 71) TNBC to determine if the difference in PD-L1 positivity correlated with clinicopathological or genomic characteristics (Table 2). There was no difference in age, sex, or genetic ancestry between the 2 groups. TNBC tissue samples from the breast did have an observed enrichment for PD-L1 positivity compared to TNBC tissue samples from a metastatic site (57% vs. 44%), but this was not statistically significant (*P* = .1766). The most common specimen sites are breast, lymph node, liver, skin, and lung representing 44%, 20%, 8%, 6%, and 5% of the entire TNBC cohort, respectively. PD-L1 positivity rates varied by metastatic site, highest in the lung and lymph nodes and lowest in the liver (Table 3). Median TMB and the rate of TMB ≥10 mut/MB were similar, and no patients had MSI-H status (Table 2). The landscape of GA was also similar between the PD-L1(+) and the PD-L1(−) cohorts. There were no significant differences in the frequency of the top of 30 most frequently altered genes of either group (Table 4). GA in *TP53*, *MYC*, *RAD21*, *PIK3CA*, and *PTEN* was most prevalent in the overall cohort. The frequency of the targetable genes *BRCA1* and *BRCA2* were also similar (7% vs. 10% and 8% vs. 3%, respectively). Amplification of *CD274* was seen in 6 PD-L1 (+) cases (8%) and only 1 PD-L1(−) case (1%) (*P* = .12).

**Table 2.** Comparison of PDL1+ and PDL1− TNBC using DAKO 22C3 PD-L1 IHC assay.

	PD-L1+ (*N* = 71)	PD-L1− (*N* = 71)	*P*-value
Age, median (range) years	57 (31-89+)	61 (32-88)	.124
Sex (% female)	71 (100%)	71 (100%)	1
Specimen site, breast/metastasis	36/35 (51%/49%)	27/44 (38%/62%)	.1766
Genetic ancestry (%)			.6003
AFR	14 (20%)	19 (27%)	
AMR	7 (10%)	8 (11%)	
EAS	2 (3%)	1 (1%)	
EUR	46 (65%)	43 (61%)	
SAS	2 (3%)	0 (0%)	
TMB, median [IQR] mut/Mb	2.5 [1.25-5]	2.5 [2.5-5.0]	.764
TMB ≥10 mut/Mb	6 (8%)	3 (4%)	.4934
MSI-H	0	0	1
gLOH	21.0 [12.0-26.2]	15.2 [7.4-26.1]	.1486

Abbreviations: AFR, African; AMR, Central and South American; CPS, combined positive score; EUR, European; gLOH, genomic loss of heterozygosity; PD-L1, programmed death ligand-1; SAS, South Asian; MSI-H, microsatellite instability-High; TMB, tumor mutational burden.

**Table 3.** Rate of PD-L1 positivity by specimen site of tested tissue in TNBC using DAKO 22C3 PD-L1 IHC assay.

Specimen site	PD-L1 +	Total
Lung	5 (71%)	7
Lymph node	19 (66%)	29
Breast	36 (57%)	63
Abdominal wall	1 (50%)	2
Pleura	1 (50%)	2
Skin	4 (44%)	9
Chest wall	2 (40%)	5
Soft tissue	1 (33%)	3
Liver	2 (17%)	12
Brain	0 (0%)	3
Bone	0 (0%)	2
Spine	0 (0%)	1
Pelvis	0 (0%)	1
Trachea	0 (0%)	1
Head and neck	0 (0%)	1
Bone marrow	0 (0%)	1

**Table 4.** Frequency of gene alterations in TNBC by PD-L1 status using DAKO 22C3 PD-L1 IHC assay.

Gene	PDL1+ (*N* = 71)	PDL1− (*N* = 71)	*P* value, corrected
*TP53*	66 (93%)	56 (79%)	.8394407
*MYC*	24 (34%)	22 (31%)	1
*RAD21*	18 (25%)	18 (25%)	1
*PIK3CA*	19 (27%)	16 (23%)	1
*PTEN*	11 (15%)	18 (25%)	1
*RB1*	10 (14%)	11 (15%)	1
*CDKN2A*	10 (14%)	7 (10%)	1
*FGFR1*	9 (13%)	7 (10%)	1
*WHSC1L1*	8 (11%)	8 (11%)	1
*LYN*	8 (11%)	5 (7%)	1
*ZNF703*	7 (10%)	6 (8%)	1
*CDKN2B*	7 (10%)	6 (8%)	1
*CCNE1*	6 (8%)	6 (8%)	1
*CCND1*	6 (8%)	6 (8%)	1
*BRCA1*	7 (10%)	5 (7%)	1
*MCL1*	6 (8%)	6 (8%)	1
*NOTCH2*	6 (8%)	6 (8%)	1
*FGF19*	5 (7%)	6 (8%)	1
*FGF3*	5 (7%)	6 (8%)	1
*PIK3R1*	3 (4%)	8 (11%)	1
*FGF4*	5 (7%)	6 (8%)	1
*CREBBP*	6 (8%)	4 (6%)	1
*KRAS*	3 (4%)	6 (8%)	1
*AKT2*	3 (4%)	6 (8%)	1
*NF1*	4 (6%)	5 (7%)	1
*JAK2*	6 (8%)	3 (4%)	1
*FGF23*	3 (4%)	5 (7%)	1
*MTAP*	3 (4%)	5 (7%)	1
*KDM5A*	4 (6%)	4 (6%)	1
*NOTCH1*	6 (8%)	2 (3%)	1

### PD-L1 Positive versus PD-L1 Negative HR+/HER2− Cohort Based on DAKO 22C3 CDx Assay

HR+/HER2− cases were also compared based on their PD-L1 expression. Patients were of similar median age and genetic ancestry (Supplementary Table S1). Most tested samples were from a metastatic site in both the PD-L1(−) and PD-L1(+) groups (69% and 70%, respectively). The most prevalently tested metastatic sites were the liver (*n* = 46), lymph node (*n* = 21), spine (*n* = 8), bone (*n* = 7), and lung (*n* = 6) with varying PD-L1 expression by the site (Supplementary Table S2). Both the rate of TMB ≥10 mut/MB and the median TMB were similar in the 2 cohorts. Median gLOH was significantly higher in the PD-L1(+) patients (17.3% vs. 9.3%, *P* < .001). In addition, differences in the frequency of several gene alterations among the top 30 altered genes most frequently found in the whole cohort were identified (Supplementary Table S3). *TP53*, *CREBBP*, and *CCNE1* alterations were more prevalent in the PD-L1(+) group compared to the PD-L1(−) group (*P* = .0027, *P* = .010, and *P* = .005, respectively).

## Discussion

In this study, we evaluated the landscape of PD-L1 IHC expression using DAKO 22C3 assay in a large cohort of breast carcinoma patients. This landscape demonstrated variable expression based on receptor subtype. TNBC had the highest median expression of PD-L1 and the greatest (approximately 50%) PD-L1 positivity rate based on the CPS ≥10 threshold per the companion diagnostic label. We also observed an approximately 15% PD-L1 positivity rate in HR+/HER2− cases. Prospective clinical trials will be needed to determine if these HR+/HER2− patients will benefit from ICPI therapy and what the optimal cut-off is to predict benefit.

We also saw that the PD-L1(+) and PD-L1(−)TNBC groups were similar in both clinicopathologic and genomic characteristics. Patients were of similar age distributions, and there was a similar distribution of genetic ancestry. We also examined the prevalence of other ICPI biomarkers. Median TMB was similar in both groups and there was no significant difference between the PD-L1(+) and PD-L1(−) groups in the percent of patients with TMB ≥10 mut/Mb. The landscape of concurrent alterations also showed no differences. These results demonstrate that these other characteristics cannot be used as surrogates to predict whether PD-L1 expression is likely for TNBC patients and that evaluation of PD-L1 by immunohistochemistry is necessary to properly evaluate this biomarker for patients in the clinic and clinical trials. Of note, these results differed from studies in non-small cell lung cancer, urothelial carcinoma, and cervical carcinoma where the authors found significant clinical and molecular differences between the PD-L1(+) and PD-L1(−) groups.^35-37^

We noticed that TNBC tissue samples from the breast had a higher rate of PD-L1 positivity compared to TNBC tissue samples from a metastatic site (57% vs. 44%), though it did not reach statistical significance. This is consistent with a prior study that also showed lower PD-L1 expression in metastatic lesions and substantial variability in PD-L1 positivity rates by metastatic site.^38^ Taken together, these observations suggest that the immune microenvironment of metastatic lesions may differ from that of primary tissues in TNBC as hypothesized by other studies of the immune landscape specific to metastases.^39-42^ This data have important implications for the specimen sites to submit for testing and clinical trial design.

In the HR+/HER2− cohort, PD-L1 positivity did correlate with other genomic differences. Median gLOH was higher in the PD-L1(+) subgroup, and alterations in *TP53*, *CREBBP*, and *CCNE1* were also enriched in this population. The elevated gLOH in the PD-L1(+) could provide a rationale for possible therapy combinations of poly (ADP-ribose) polymerase (PARP) inhibitors and immunotherapy for HR+ disease. *TP53* has been shown to be enriched in metastatic HR+ breast cancer, but the interaction between *TP53* alterations, PD-L1 expression, and prognosis merits further study based on these results.^43^

Notably, nearly a quarter of TNBC patients in this study were of predominantly African ancestry regardless of PD-L1 expression. This finding is consistent with reports that the TNBC phenotype is enriched in African American patients.^6,7^ Similar PD-L1 expression and highly comparable immune microenvironment between TNBC in African American and Caucasian patients have also been shown earlier.^41^ It is regrettable that patients identifying as being of Black race made up only 7% of enrollees in the IMpassion130 trial and only 4% of those enrolled in Keynote-355.^10,19^ Given this particular unmet need of African American patients and the evidence that PD-L1 expression is prevalent in this population, future clinical trials assessing PD-L1 as a biomarker of response to ICPI should aim to increase enrollment of these patients.

One limitation of this study is the lack of treatment history for the included patients. It is unknown if patients received prior therapy including hormone therapy and chemotherapy which could possibly have altered the clonal evolution and genomics of the tumor. The study is retrospective in nature, and prospective clinical trials will be needed to test further hypotheses in the active treatment of patients.

## Conclusion

The subtypes of breast cancer have distinct patterns of PD-L1 expression, and thus investigations into the efficacy of ICPI in non-TNBC patients may consider including analysis of optimum cutoffs for non-TNBC patients. In TNBC, PD-L1 positivity is not associated with other clinicopathologic or genomic features and thus should continue to be integrated into future studies of the immune microenvironment and immunotherapy efficacy. TNBC tissue samples from the breast did have an observed but not statistically significant enrichment for PD-L1 positivity compared to TNBC tissue samples from a metastatic site, a finding that merits future research in a larger cohort.

## Supplementary Material

oyad025_suppl_Supplementary_Tables

## Data Availability

The data underlying this article are available in the article and in its online supplementary material.

## Abbreviations

AFR: African
AMR: Central and South American
CGP: comprehensive genomic profiling
CPS: combined positive score
EAS: East Asian
ER: estrogen receptor
EUR: European
FFPE: formalin-fixed paraffin-embedded
gLOH: genomic loss of heterozygosity
HER2: human epidermal growth factor receptor
ICPI: immune checkpoint inhibitor
Mb: megabases
MSI: microsatellite instability
OS: overall survival
PD-1: programmed death receptor-1
PD-L1: programmed death ligand-1
PR: progesterone receptor
SAS: South Asian
TILs: tumor-infiltrating leukocytes
TMB: tumor mutational burden
TMB-High: tumor mutational burden ≥10 mutations per megabase
TNBC: triple-negative breast cancer
