# Supplementary material for: Variable Landscape of PD-L1 Expression in Breast Carcinoma as Detected by the DAKO 22C3 Immunohistochemistry Assay
Source: Oncologist. 2023 Mar 2;28(4):319–26. doi: 10.1093/oncolo/oyad025 (PMC10078903; doi:10.1093/oncolo/oyad025)
Supplement: oyad025_suppl_Supplementary_Tables [file oyad025_suppl_supplementary_tables.docx]

| **Supplementary Table 1**. Comparison of PDL1+ and PDL1- HR+/HER2- breast cancer utilizing DAKO 22C3 PD-L1 IHC assay | | |  |
| --- | --- | --- | --- |
|  | **PD-L1 +** | **PD-L1 -** | **p value** |
| **N** | 26 | 142 |  |
| **Age, median (range) yrs** | 64 (39-89+) | 62 (29-84) | 0.552 |
| **Sex (% Female)** | 26 (100%) | 142 (100%) | 1 |
| **Specimen site, breast/metastasis** | 8/18 (31%/69%) | 42/100 (30%/70%) | 1 |
| **Genetic Ancestry (%)** |  |  | 0.518 |
| AFR | 6 (23%) | 18 (13%) |  |
| AMR | 3 (12%) | 81 (13%) |  |
| EAS | 1 (4%) | 3 (2%) |  |
| EUR | 16 (62%) | 101 (71%) |  |
| SAS | 0 (0%) | 2 (1%) |  |
| **TMB, median [IQR] mut/Mb** | 3.1 [1.25-5.9] | 2.5 [1.25-5.0] | 0.618 |
| **TMB ≥10 mut/Mb** | 3 (12%) | 13 (9%) | 0.717 |
| **MSI-H** | 0 | 0 | 1 |
| **gLOH** | 17.3 [14.3-22.3] | 9.3 [5.5-15.2] | <0.001 |

**Supplementary Table 2.** Rate of PD-L1 positivity by specimen site of tested tissue in HR+/HER2- breast cancer utilizing DAKO 22C3 PD-L1 IHC assay

| **Specimen Site** | **PD-L1 +** | **Total** |
| --- | --- | --- |
| Breast | 8 (16%) | 50 |
| Liver | 7 (15%) | 46 |
| Lymph Node | 7 (33%) | 21 |
| Spine | 0 (0%) | 8 |
| Bone | 0 (0%) | 7 |
| Lung | 1 (17%) | 6 |
| Skin | 0 (0%) | 5 |
| Soft Tissue | 1 (20%) | 5 |
| Pleura | 0 (0%) | 5 |
| Chest Wall | 0 (0%) | 3 |
| Omentum | 0 (0%) | 2 |
| Brain | 2 (100%) | 2 |
| Pelvis | 0 (0%) | 2 |
| Diaphragm | 0 (0%) | 1 |
| Colon | 0 (0%) | 1 |
| Eye | 0 (0%) | 1 |
| Uterus | 0 (0%) | 1 |
| Peritoneum | 0 (0%) | 1 |
| Mediastinum | 0 (0%) | 1 |

**Supplementary Table 3.** Frequency of gene alterations in HR+/HER2- breast cancer by PD-L1 status utilizing DAKO 22C3 PD-L1 IHC assay

| **Gene** | **PDL1- (N=71)** | **PDL1+ (N=71)** | **P value, corrected** |
| --- | --- | --- | --- |
| *PIK3CA* | 9 (35%) | 61 (43%) | 0.865 |
| *TP53* | 19 (73%) | 43 (30%) | 0.00265 |
| *CCND1* | 5 (19%) | 36 (25%) | 0.905 |
| *FGF3* | 5 (19%) | 32 (23%) | 0.963 |
| *FGF4* | 5 (19%) | 31 (22%) | 1 |
| *FGF19* | 5 (19%) | 31 (22%) | 1 |
| *ZNF703* | 7 (27%) | 29 (20%) | 0.785 |
| *GATA3* | 2 (8%) | 29 (20%) | 0.569 |
| *WHSC1L1* | 6 (23%) | 26 (18%) | 0.905 |
| *FGFR1* | 6 (23%) | 24 (17%) | 0.782 |
| *ESR1* | 2 (8%) | 24 (17%) | 0.751 |
| *RAD21* | 5 (19%) | 23 (16%) | 0.963 |
| *CDH1* | 1 (4%) | 22 (15%) | 0.583 |
| *PTEN* | 3 (12%) | 15 (11%) | 1 |
| *ARID1A* | 3 (12%) | 15 (11%) | 1 |
| *MYC* | 8 (31%) | 14 (10%) | 0.062 |
| *BRCA2* | 0 (0%) | 14 (10%) | 0.559 |
| *MAP2K4* | 0 (0%) | 13 (9%) | 0.583 |
| *EMSY* | 1 (4%) | 12 (8%) | 0.905 |
| *MAP3K1* | 1 (4%) | 12 (8%) | 0.905 |
| *FGFR2* | 2 (8%) | 12 (8%) | 1 |
| *AKT1* | 2 (8%) | 9 (6%) | 0.905 |
| *CREBBP* | 5 (19%) | 2 (1%) | 0.01048 |
| *CCNE1* | 5 (19%) | 1 (1%) | 0.00503 |
| *NFKBIA* | 3 (12%) | 1 (1%) | 0.0720 |
| *KIT* | 2 (8%) | 1 (1%) | 0.314 |
| *BRCA1* | 2 (8%) | 4 (3%) | 0.583 |
| *ZNF217* | 2 (8%) | 5 (4%) | 0.683 |
| *ESR1* | 2 (8%) | 24 (17%) | 0.751 |
| *GATA3* | 2 (8%) | 29 (20%) | 0.569 |
